# Time trends of esophageal and gastric cancer mortality in China, 1991–2009: an age-period-cohort analysis

**DOI:** 10.1038/s41598-017-07071-5

**Published:** 2017-07-28

**Authors:** Mengmeng Li, Xia Wan, Yanhong Wang, Yuanyuan Sun, Gonghuan Yang, Li Wang

**Affiliations:** Department of Epidemiology and Biostatistics, Institute of Basic Medical Sciences, Chinese Academy of Medical Sciences; School of Basic Medicine, Peking Union Medical College, Beijing, China

## Abstract

Esophageal and gastric cancers share some risk factors. This study aimed to compare the long-term trends in mortality rates of esophageal and gastric cancers in China to provide evidence for cancer prevention and control. Mortality data were derived from 103 continuous points of the Disease Surveillance Points system during 1991–2009, stratified by gender and urban-rural locations. Age-period-cohort models were used to disentangle the time trends of esophageal and gastric cancer mortality. The downward slope of the period effect for esophageal cancer was steeper than that for gastric cancer in rural areas. Cohort effect patterns were similar between esophageal and gastric cancers, with an inverse U-shape peaking around the late 1920s and early 1930s. A second peak, appearing around the 1950s, was weaker than the first but apparent in males, especially for esophageal cancer. The more marked changes in period effect for esophageal cancer in rural areas suggest esophageal cancer screening practices are effective in reducing mortality, and similar programs targeting gastric cancer should be implemented. The similarities of the cohort effects in these two cancers support the implication of nutrition deficiency in early childhood in the development of upper gastrointestinal cancer.

## Introduction

Upper gastrointestinal cancers, usually referred to as esophageal cancer and gastric cancer, are among the leading causes of cancer mortality around the world, accounting for 13.7% of the total cancer deaths^1^. As a high-risk area for digestive cancers, China was estimated to suffer 375,000 esophageal cancer deaths and 498,000 gastric cancer deaths in 2015, which together represented 31.0% of all cancer deaths^2^. In the Chinese cancer profile, gastric cancer and esophageal cancer ranked third and fourth in cancer mortality in 2012, with mortalities reaching as high as 22.04 and 15.58 per 100,000, respectively^3^.

Esophageal and gastric cancers share some common risk factors, including low intake of fruits and vegetables, tobacco smoking and alcohol consumption^4–6^, although the relative importance depends on the cancer type. The longitudinal trends of upper gastrointestinal cancer mortality could be linked to variations in these shared and other cancer-specific risk factors, or to the implementation of screening and treatment practices. Thus, comparing similarities and differences in long-term trends between esophageal and gastric cancers can help identify factors contributing to these trends and direct future efforts for cancer prevention and control.

Overall, esophageal and gastric cancers have declined in China. However, overall trends may mask important differences in the cancer death data^7, 8^, which may veil underlying causes. In order to identify the causes of trends in mortality, it is necessary to distinguish between period and cohort patterns. Period patterns suggest effects of factors that influence all age groups simultaneously, whereas cohort patterns may indicate long-lasting effects of factors happening in early life circumstances. By disentangling the overall trends under the Age-Period-Cohort (APC) framework, we can obtain a more comprehensive understanding of the driving forces behind them. Therefore, the aim of this study was to compare the time trends of esophageal and gastric cancer mortality with an age-period-cohort analysis, using a relatively fixed population from Disease Surveillance Points system (DSPs) in 1991–2009 in China.

## Results

### Mortality rates of upper gastrointestinal cancer

A total of 49,672 esophageal cancer deaths and 79,105 gastric cancer deaths were reported by 103 continuous disease surveillance points after 388,646,789 person years’ follow-up (Table S1). Gastric cancer deaths occurred more frequently than esophageal cancer for all four groups. Both esophageal and gastric cancer deaths occurred more than twice as frequently in men than in women. The highest mortality rates were seen in rural males, with age-standardized mortality rates (ASMRs) of 21.89 and 31.00 per 100,000 in 2006–2009 for esophageal cancer and gastric cancer, respectively.

### Age-specific mortality rates by periods

**Figure 1:**
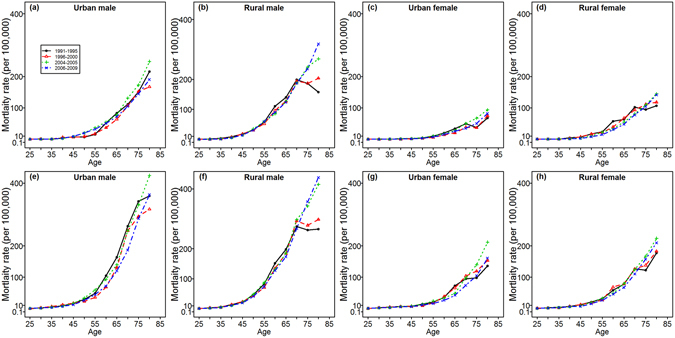
Age-specific mortality rates of esophageal and gastric cancers by period of death, stratified by region and sex during the period of 1991–2009. (**a–d**) In the first row represent esophageal cancer mortality; (**e–h**) In the second row represent gastric cancer mortality.

Both esophageal and gastric cancer death rates showed rapid increases after the age of 45, with a faster increase for gastric cancer. Age-specific death rates for both cancers declined from 1991–1995 to 2006–2009 for the youth and those in middle age, but increased after the age of 70. An exception was found in esophageal cancer for urban males, with no obvious decrease among the 45–59 age group. The mortality rates were relatively higher in 2004–2005 than in other periods, partly because the data from this period were actively collected in a national survey and not by passive surveillance as in other years. The variations in the age-specific mortality rates for esophageal and gastric cancers across time periods suggested the existence of a cohort effect (Fig. 1).

### Age-specific mortality rates by birth cohort

**Figure 2:**
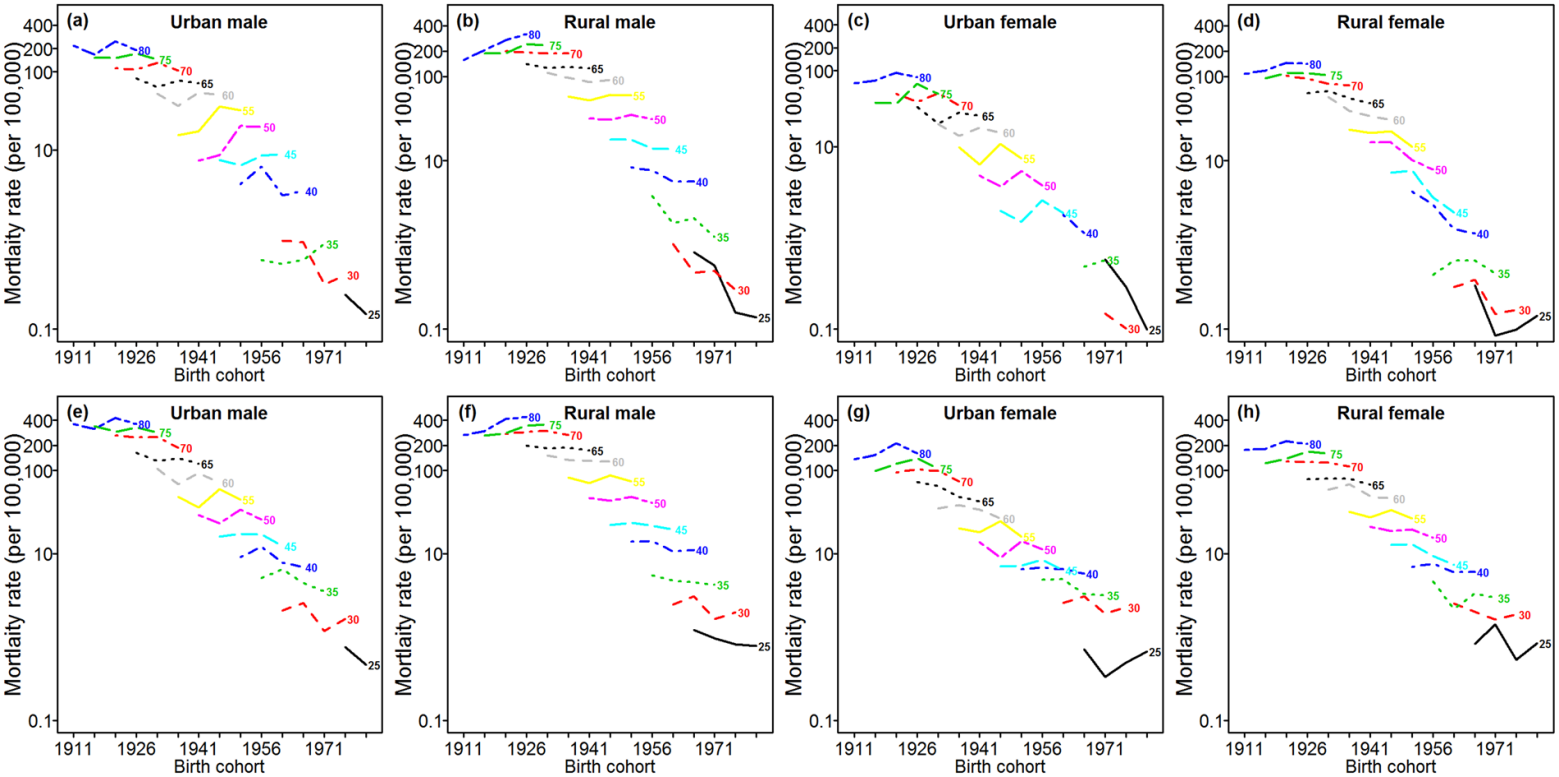
Cohort-specific mortality rates of esophageal and gastric cancers on a log scale in different age groups, stratified by region and sex during the period of 1991–2009. (**a–d**) In the first row represent esophageal cancer mortality; (**e–h**) in the second row represent gastric cancer mortality.

For esophageal and gastric cancers, age-specific rates decreased consistently by birth cohort with the exception of the elderly. The mortality rates for those older than 75 years increased with birth cohort for urban females, rural males and rural females (Fig. 2b–d, f–h). A notable phenomenon of esophageal cancer is the upward trend for those aged between 45 and 64, especially for the 50 to 59 year-old age groups for urban males (Fig. 2a). The nonparallelism among the age curves by birth cohort indicates the existence of a period effect.

### Age-period-cohort effects on upper gastrointestinal cancer

**Figure 3:**
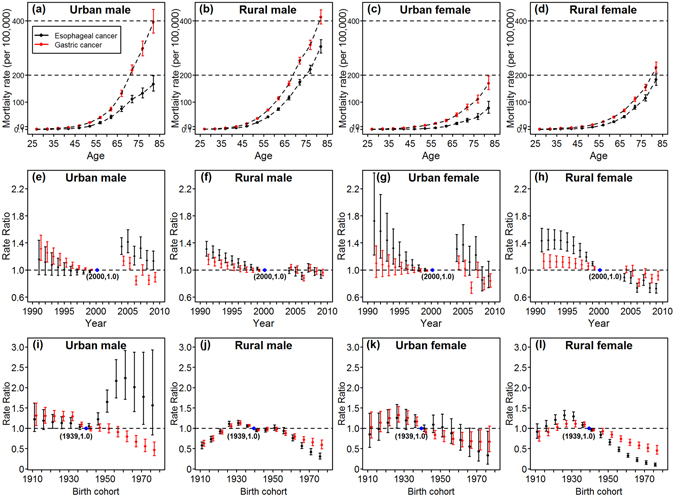
Age, period, and cohort effects on esophageal cancer and gastric cancer mortality, stratified by region and sex. (**a–d**) In the first row represent age-specific mortality rates in the reference period of 2000 after adjusting for period and cohort effects. (**e–h**) In the second row are the estimated period effects, and the blue dot is the reference period. (**i–l**) In the last row are the cohort effects, and the blue dot is the reference cohort.

We chose the full APC models based on the goodness of fit of the sub-models (Table 1), and the model-derived age, period and cohort effects are shown in Fig. 3 for men and women in urban and rural areas.

**Table 1:** Akaike information criterion (AIC) of age-period-cohort sub-models for upper gastrointestinal cancer mortality, China, 1991–2009.

Sub-models	Esophageal cancer				Gastric cancer			
	Urban male	Urban female	Rural male	Rural female	Urban male	Urban female	Rural male	Rural female
Age-period-cohort	1013	780.4	1363	1201	1190	1121	1531	1383
Age-drift	1129	851.9	1609	1394	1346	1238	1560	1514
Age-period	1060	781.2	1580	1361	1206	1131	1722	1457
Age-cohort	1091	842.7	1404	1227	1323	1221	1622	1434

After controlling for period and cohort effects, similar age patterns were observed between esophageal and gastric cancers, with mortality rates increasing exponentially with age. The upward slope was sharper for gastric cancer. Males and people in rural areas showed steeper increases with age than their counterparts (Fig. 3a–d).

The downward-sloping curves show that period effects slightly contributed to the decline in esophageal and gastric cancer mortality. In rural areas, the slope of the period effects was steeper for esophageal cancer than for gastric cancer. The downward trends were slower in males for esophageal cancer and in rural areas for gastric cancer. A sudden increase in 2004 was captured by the period effect (Fig. 3e–h).

The cohort effects were more pronounced than the period effects. The cohort effect patterns were similar between esophageal cancer and gastric cancer, with an inverse U-shape (Fig. 3i–l) peaking around the late 1920s and early 1930s (Table 2). For urban males, there was no peak around 1930, and a slight but steady decrease since 1911 could be observed. However, this trend accelerated slightly after the 1930 birth cohort (Fig. 3i). A second peak appeared around the 1950s, which was weaker than the first but apparent in males. The risk of esophageal cancer death for urban males was highest for those born in 1959, with rate ratio (RR) being 2.26 (95% CI: 1.77, 2.88) compared with the 1939 reference cohort. Accelerating and continuous declines occurred in subsequent generations. The estimated annual changes (net drift due to the linear period and cohort effect) were slower in men for esophageal cancer and in rural areas for gastric cancer (Table S2).

**Table 2:** Highest cohort effects (rate ratio, RR) on esophageal and gastric cancer mortality rates and the corresponding birth cohorts, compared with the 1939 birth cohort.

	Esophageal cancer		Gastric cancer	
	Birth cohort	RR (95%CI)	Birth cohort	RR (95%CI)
Urban male	1959	2.26 (1.77,2.88)	1911	1.31 (1.07,1.62)
Rural male	1929	1.15 (1.09,1.21)	1931	1.13 (1.08,1.18)
Urban female	1927	1.26 (1.02,1.57)	1927	1.33 (1.16,1.53)
Rural female	1928	1.34 (1.23,1.45)	1934	1.12 (1.06,1.19)

## Discussion

This study used the DSPs to provide a comprehensive comparison between the mortality trends of esophageal and gastric cancers in China. The major findings show that the upper gastrointestinal cancer mortality increased exponentially with age, the rate of which was faster for gastric cancer than esophageal cancer. The downward period effect was more favorable for esophageal cancer in rural areas than for gastric cancer. An inverse U-shaped cohort effect was found for both esophageal and gastric cancers, and those born in the 1920s or 1930s were at the highest risk. Another peak was observed in the late 1950s in men, especially for esophageal cancer.

The similarity of the cohort patterns between esophageal and gastric cancers indicate that shared risk factors may contribute to the long-term trend, primarily factors such as tobacco smoking and diet^4–6^. Another study in Spain also reported that the shapes of the cohort effect were similar among upper gastrointestinal cancers^9^. An inverse U-shaped cohort effect for gastric cancer was also confirmed in Denmark, England, Switzerland, Italy, Japan and the United States^10^, the downward trend of which began in the 19th century, much earlier than in China, suggesting that the shared risk factors of upper gastrointestinal cancers are closely related to socioeconomic and hygienic conditions. Given the worldwide epidemic of smoking in the 20th century, it may not be directly responsible for the downward trend. Dietary behavior is probably the driving force, including improved nutrition and availability of fresh fruits and vegetables, as well as reduced intake of salted and pickled food, especially after the introduction of refrigeration^4, 11, 12^.

A notable phenomenon is that the highest risk for upper gastrointestinal cancers was observed among those born in the late 1920s and early 1930s. Similar results were confirmed using data from the Ministry of Health Vital Registration system^13^. One possible explanation is that several natural disasters have occurred during that period, followed by severe famine and deaths^14^. The newborns who survived those famines may have suffered from malnutrition during infancy. Fruits and vegetables can protect against both esophageal and gastric cancers, and zinc intake has also been shown to reduce the risk for those cancers in the Asian population^15^. Foods containing beta-carotene, vitamin C, folate, pyridoxine, and vitamin E are negatively associated with esophageal cancer, and foods containing selenium is considered to be protective against gastric cancer^16^. A 10-year follow-up of the General Population Nutrition Intervention Trial conducted in Linxian indicated that vitamin and mineral supplementation were protective against gastric cancer mortality and esophageal cancer deaths if the intervention was taken younger than 55 years^17^. The deficiency of those food and nutritional elements during a famine period may contribute to the increase of risk in early generations. Another explanation for gastric cancer is that nutrition in childhood can interact with *Helicobacter pylori* (*H. pylori)* infection. In malnutrition conditions, the resulting low acid secretion would increase the susceptibility to the development of *H. pylori*-induced atrophic gastritis and gastric cancer^18^.

Another peak in the cohort effect occurred in the late 1950s. Those who were born in that period experienced the Great Famine in China, which further supported the role of nutrition deficiency in the etiology of upper gastrointestinal cancer. A previous study also found that the risk of gastric cancer mortality in Zhaoyuan county population increased during the Chinese Great Famine period^19^. However, the cohort effect was much higher for esophageal cancer in urban males, which was in line with another study that used incidence data in Shanghai, China^20^. The particularly high effect for this group suggests that some other unknown factors may interact with famine exposure.

The other divergence of esophageal and gastric cancers in cohort effects may be due to the mixed effect of shared and cancer-specific risk factors. The relative importance of shared risk factors depends on cancer types. For example, the summary relative risks of various fruits and vegetables are different among upper gastrointestinal cancer, and the protective effects seem larger against esophageal cancer than gastric cancer^4^. Smoking was shown to be a much higher risk factor for esophageal squamous cell carcinoma (ESCC) than other types of upper gastrointestinal cancer in a prospective study, and the population attributable risk proportion of ever smoking is 77% (95% CI: 0.55, 0.89) for ESCC^21^. With regard to the cancer-specific risk factors, gastric cancer is also caused by *H. pylori* infection and high salt intake^4, 22^. As to the different histological type of esophageal cancer, esophageal adenocarcinoma (EAC) is associated with gastroesophageal reflux disease and obesity^23, 24^, while alcohol and hot beverages play an important role in the etiology of ESCC^4, 25^, which is the main type in Asia.

The downward trend of the period effect is faster for esophageal cancer than gastric cancer, which may be partly due to the endoscopy screening program. The age-standardized five-year relative survival rate for patients in China diagnosed in 2003–2005 is 20.9% for esophageal cancer and 27.4% for gastric cancer^26^. The poor survival is primarily due to the fact that most patients are diagnosed and treated at late stages. The five-year survival rates of early-stage esophageal and gastric cancers can reach as high as 86.14% and 93.2%, respectively^27, 28^, but can be very low at advanced stages. Patients with early-stage upper gastrointestinal cancer are usually asymptomatic; thus, the development of more effective screening methods and the practice of screening in high-risk areas may have contributed to reductions in cancer-associated mortality^29^. Several extensive mass screening programs have recently been conducted in some rural areas in China for esophageal carcinomas, but not for gastric cancer^30^, which may partly explain the faster downward period effect for esophageal cancer, especially in rural areas. Fortunately, gastric cancer has gradually begun to be included in screening programs. In 2005, the Ministry of Health of China launched an Early Detection and Treatment Program. The first aim of this program was to detect cervical cancer and esophageal cancer, and the program gradually expanded to six cancers including gastric cancer^30^. Since 2008, the Early Detection and Treatment Program has included the high-risk Huai River basin, where it mainly targets digestive cancers. The Early Detection and Treatment Program were initiated in cities since 2012 and expanded to 16 provinces in 2015, and upper gastrointestinal cancers are among the five screened cancers. Further studies are needed to evaluate the period effects of these programs on esophageal and gastric cancers.

A huge gap still exists between urban and rural areas in China with regard to upper gastrointestinal cancer mortality, especially gastric cancer. The ASMR in rural areas far exceeded that in urban areas during the entire study period. With extensive screening programs, the annual percent change for esophageal cancer is larger in rural areas, and the survival rate has become nearly equal between rural and urban areas, with age-standardized 5-year relative survival rates for patients diagnosed in 2003–2005 of 21.2% and 19.1%, respectively^26^. However, the gastric cancer mortality declined slower in rural areas, and the 5-year survival rate in rural areas (24.9%) is far below that in urban areas (32.5%)^26^. In addition to the inadequacy of mass screening, early detection and treatment programs for gastric cancer, the poor access to cancer care for rural migrant workers also contributes to the health disparity in cancer mortality^31^.

The gender difference in mortality levels partly reflects the biological susceptibility to upper gastrointestinal cancer, as well as the different exposures to risk factors. The annual percent changes of upper gastrointestinal cancers are lower in men than women, especially for esophageal cancer. Alcohol is closely associated with an increased risk of ESCC, but not of EAC, cardia gastric cancer or non-cardia gastric cancer^4^. The interaction between alcohol consumption and cigarette smoking amplifies this effect^25^. Considering the high exposure of smoking and drinking in men and the harmful impact on esophageal cancer, primary prevention measures are needed to reduce the exposure.

The strength of our study is that data from 103 continuous disease surveillance points were used in the trend analysis. Obvious spatial clustering and geographical variation was seen in esophageal and gastric cancer mortality, and the mortality levels varied among surveillance areas^32^. Changes in the surveillance points may cause fluctuations in the true underlying trend.

Our study has several limitations. First, the third national retrospective mortality survey was conducted in 2005 to collect causes of death in 2004 and 2005 based on the sampling sites in the DSPs. Thus, our data consist of two parts: active survey data in 2004–2005 and passive surveillance reports in the other years. This may cause fluctuation in the trends. However, this phenomenon has been captured and controlled by period effects, which represent the forces that influence all age groups. Second, the DSPs went through a sampling site expansion and adjustment in 2005. Although 103 points were reserved and continuous throughout the study period, the coverage of these sites substantially increased, making the mortality rates more stable in later years. The age and sex structure of the covered population, however, did not change much before and after the expansion. Thus the mortality rates are still comparable. Third, due to the unavailability of the subtype data of esophageal and gastric cancers from the DSPs system, we did not analyze esophageal and gastric cancer trends by subtypes, which can give more explicit implications about the etiological changes. For example, the diverging trends of EAC and ESCC have been observed in some western countries. The incidence of EAC has increased probably due to obesity^33^. In China, the prevalence of obesity in children and adults has increased during the past three decades^34, 35^, which may lead to the increased trend of EAC mortality. Although ESCC accounts for 91.61% of all esophageal cancer according to the annual report on status of cancer in China^36^, changes in other subtypes should also be cautioned. Future work is needed to ensure the accurate diagnosis and recording of cancer subtypes in surveillance data.

In conclusion, our analysis shows that age, period and cohort effects account for the similarity and divergence in mortality trends of esophageal and gastric cancers. The favorable downward period effect for esophageal cancer highlights the benefit of screening practices. The cohort effects indicate the role of nutrition deficiency in cancer etiology. However, the particularly high risk of esophageal cancer for urban males during the late 1950s suggests that some other unknown factors may interact with famine exposure, which warrants further investigation.

## Materials and Methods

### Data sources

**Figure 4:**
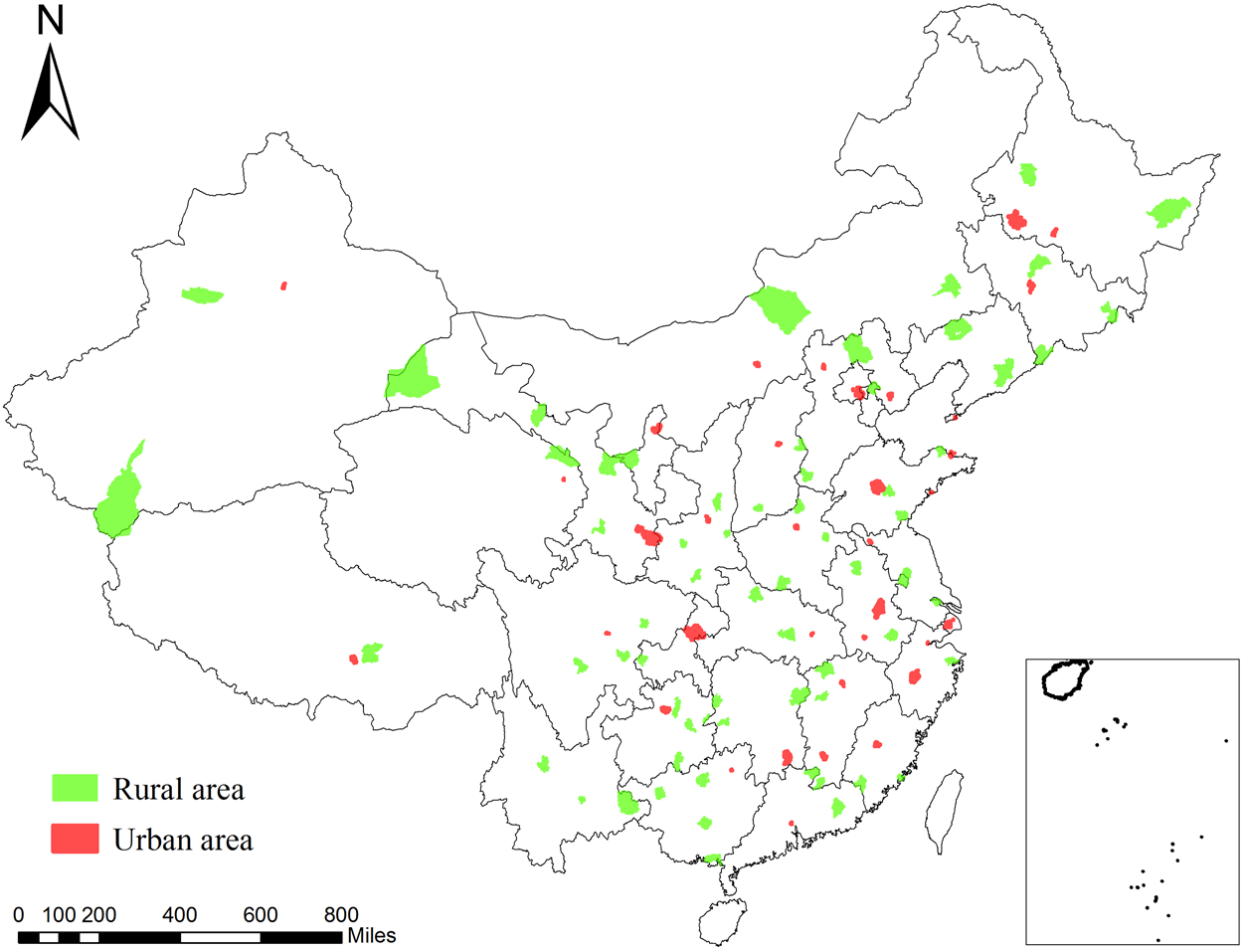
Locations of the 103 continuous disease surveillance points by urban and rural status in China. This map was generated by ArcGIS software, version 10.2 (http://www.esri.com).

DSPs system is a national mortality surveillance system that was established in China in 1990, covering 10 million populations in 145 locations by using a multi-staged stratified cluster sampling strategy. The system was expanded to 161 sites in 2005 to cover 71.4 million people^37, 38^. Despite this expansion in coverage, 103 sites were consistent during the whole period. Also, the system ceased nearly all operations from 2001 to 2003 because of DSPs adjustment. Thus, we only obtained esophageal and gastric cancer mortality data from the 103 continuous sites from 1991 to 2000 and from 2004 to 2009. For the 103 sites, random samples of rural counties or urban districts were covered during 1991–2000, while after 2004, the whole counties or districts were included. The location of the continuous points by urban and rural status across China can be found in Fig. 4.

Causes of death were coded using the International Classification of Diseases, 9th Revision (ICD-9) before 2001 and 10th Revision (ICD-10) after 2001. Esophageal cancer (ICD9: 150; ICD10: C15.0-C15.9) and gastric cancer (ICD9: 151; ICD10: C16.0-C16.9) deaths and covered populations were obtained for each surveillance point. Our study did not involve interaction with human subjects or personal identifying information, so ethical approval and informed consent were not necessary.

### Statistical analysis

The mortality data from 2004 to 2005 were verified by the third National Death Sampling Retrospective Survey. Causes of deaths in the rest of the years were recorded annually, with periodic evaluations for completeness of registration, thus we adjusted the mortality rate by under-reporting rate. The under-reporting rates come from completeness surveys carried out by the Chinese Academy of Preventive Medicine, and the detailed design and methods were described elsewhere^37, 39^.

The crude mortality rates (CMRs) were calculated for urban males, rural males, urban females, and rural females in four periods: 1991–1995, 1996–2000, 2004–2005, and 2006–2009. Adjusted mortality rates (AMRs) were calculated by adjusting under-reporting rates. Then, ASMRs were calculated using the direct method based on the 1960 world standard population (Segi 1960).

An APC analysis was used to investigate the effects of the three time variables^40, 41^. Analyses were restricted to cases aged 25 to 84 years and was grouped by 5-year age intervals. First, we visually detected age, period and cohort effects using a graphical method. We reassembled data from the aforementioned four periods with five-year age intervals in a Lexis diagram. Birth cohorts were then obtained by subtracting age from period and represented as the midpoint of the birth years. For example, those who died of upper gastrointestinal cancers during the period 1991–1995 at the age of 50–54 were born between 1937 and 1945, so the midpoint, 1941, was used to represent their birth cohort. Age-specific mortality rates were then plotted by period and birth cohort for both genders in urban and rural areas.

Second, APC models^42^ were applied to disentangle their separate effects on esophageal and gastric cancer mortality trends from 1991 to 2009. To make full use of the data, we further tabulate the data with 1-year interval for period and 5-year intervals for age. Birth cohorts were calculated using the methods described above. A Poisson regression model was estimated for both sexes in rural and urban areas, with the mortality rate observed for each age group, calendar year of death and birth cohort as the dependent variable.

$$\mathrm{log}(r(a,p))=f(a)+g(p)+h(c)$$

where f(a), g(p), h(c) represent the functions of age, period and cohort effects. To address the non-identifiability problem, we decomposed the mortality trend into an overall linear trend (drift), a non-linear period effect and non-linear cohort effect. The “drift” reflects the sum of the linear period and cohort effect^8, 42, 43^. Deviations from linearity, which are not dependent on any model constraint, were then estimated as period and cohort effects, with 1939 and 2000 as the cohort and period reference group respectively. Age effects were represented as the age-specific rates in the reference period after controlling for period and cohort effects^42^. Each term of the three functions was parameterized by natural splines with seven knots. We determined the full models a priori and sequentially fit them with age by adding drift, period and cohort variables. Goodness of fit for the sub-models were compared by the Akaike information criterion (AIC).

All data analyses were performed using the R software (version 3.0.3, R Development Core Team 2010). The *apc.fit* function in the Epi package^44^ was used to fit the age-period-cohort model.

## Electronic supplementary material

Supplementary Information
