# Supplementary material for: Time trends of esophageal and gastric cancer mortality in China, 1991–2009: an age-period-cohort analysis
Source: Sci Rep. 2017 Jul 28;7:6797. doi: 10.1038/s41598-017-07071-5 (PMC5533794; doi:10.1038/s41598-017-07071-5)
Supplement: Supplementary file 1 — Supplementary Information [file 41598_2017_7071_MOESM1_ESM.pdf]

Supplementary material

Time trends of esophageal and gastric cancer mortality in China, 1991-2009: an age-period-cohort analysis

Mengmeng Li[#], Xia Wan[#], Yanhong Wang, Yuanyuan Sun, Gonghuan Yang[*], Li Wang[*]

Department of Epidemiology and Biostatistics, Institute of Basic Medical Sciences, Chinese Academy of Medical Sciences; School of Basic Medicine, Peking Union Medical College, Beijing, China

[#]: Co-first authors

*Corresponding author at: Institute of Basic Medical Sciences, Chinese Academy of Medical Sciences and School of Basic Medicine, Peking Union Medical College, 5 Dong Dan San Tiao, Beijing 100005, China; Tel: +86-10-65288170; Email: yangghuan@vip.sina.com (Prof. Gonghuan Yang), wangli0528@vip.sina.com (Prof. Li Wang)

Table S1. Overall deaths, crude mortality rates (CMRs), adjusted mortality rates (AMRs) and age-standardized mortality rates (ASMRs) for esophageal and gastric cancer in China, 1991-2009 (/100,000)

		Person years	Esophageal cancer				Gastric cancer			
			Deaths	CMR	AMR†	ASMR*	Deaths	CMR	AMR†	ASMR*
Urban male	1991-1995	3,103,241	330	10.63	11.89	12.55	742	23.91	26.89	27.70
	1996-2000	3,658,983	362	9.89	11.61	10.54	784	21.43	25.12	22.66
	2004-2005	16,570,996	2,839	17.13	17.13	14.62	5,172	31.21	31.21	26.26
	2006-2009	37,221,143	5,374	14.44	17.17	13.25	8,970	24.10	28.71	21.71
Rural male	1991-1995	15,264,502	2,443	16.00	18.38	22.02	3,455	22.63	25.98	30.74
	1996-2000	15,468,120	2,494	16.12	18.46	20.42	3,680	23.79	27.24	30.08
	2004-2005	35,521,127	7,556	21.27	21.27	21.35	11,480	32.32	32.32	32.46
	2006-2009	71,505,277	13,553	18.95	22.89	21.89	19,265	26.94	32.56	31.00
Urban female	1991-1995	3,007,264	136	4.52	5.13	4.69	322	10.71	12.05	10.84
	1996-2000	3,559,850	126	3.54	4.17	3.42	401	11.26	13.26	10.75
	2004-2005	16,140,072	1,122	6.95	6.95	5.14	2,504	15.51	15.51	11.35
	2006-2009	36,487,642	1,875	5.14	6.07	4.09	4,186	11.47	13.58	9.11
Rural female	1991-1995	14,674,419	1,261	8.59	9.87	10.67	1,715	11.69	13.44	14.29
	1996-2000	14,837,012	1,316	8.87	10.15	10.19	1,883	12.69	14.52	14.57
	2004-2005	33,735,187	3,405	10.09	10.09	8.82	5,626	16.68	16.68	14.46
	2006-2009	67,891,954	5,480	8.07	9.74	8.09	8,920	13.14	15.87	13.11

† Adjusted for under-reporting rates

* Mortality for all age groups, age-standardized based on the 1960 world population

Table S2. The estimated annual percent change of esophageal and gastric cancer mortality rates (95%CI)

	Esophageal cancer	Gastric cancer
Urban male	0.21% (-0.35%,0.77%)	-1.67%(-2.06%,-1.28%)
Rural male	-0.08% (-0.32%,0.17%)	-0.07%(-0.27%,0.13%)
Urban female	-1.38% (-2.28%,-0.49%)	-1.91%(-2.48%,-1.33%)
Rural female	-2.03% (-2.38%,-1.68%)	-0.70%(-0.99%,-0.41%)
